# Supplementary material for: Machine learning for cell type classification from single nucleus RNA sequencing data
Source: PLoS One. 2022 Sep 23;17(9):e0275070. doi: 10.1371/journal.pone.0275070 (PMC9506651; doi:10.1371/journal.pone.0275070)
Supplement: S1 File — (PDF) [file pone.0275070.s001.pdf]

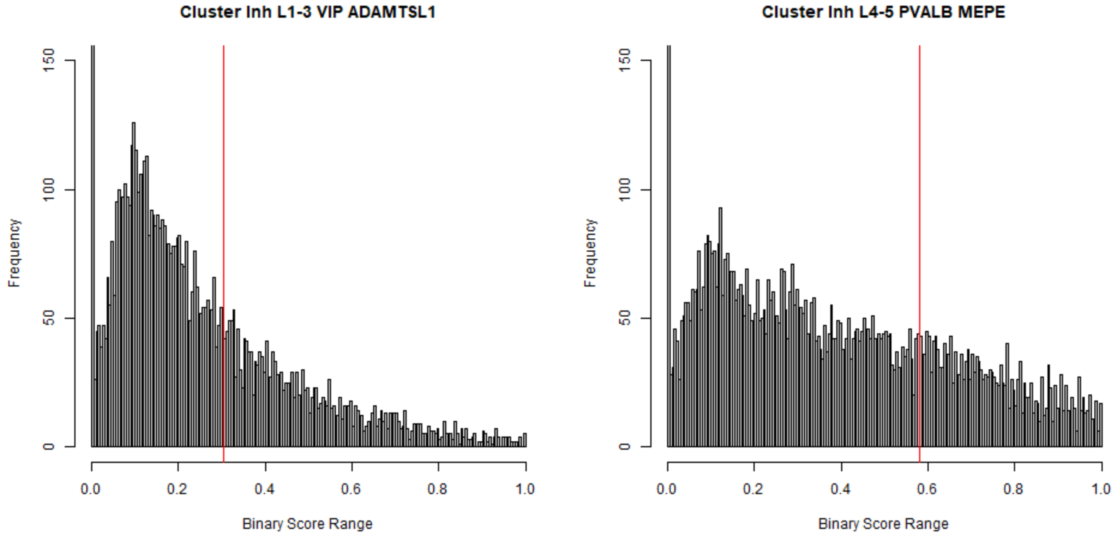

Supplementary Figure 1: Examples of Ranked Binary Scores within a Cluster. The red line displays the mean binary score for each cluster.

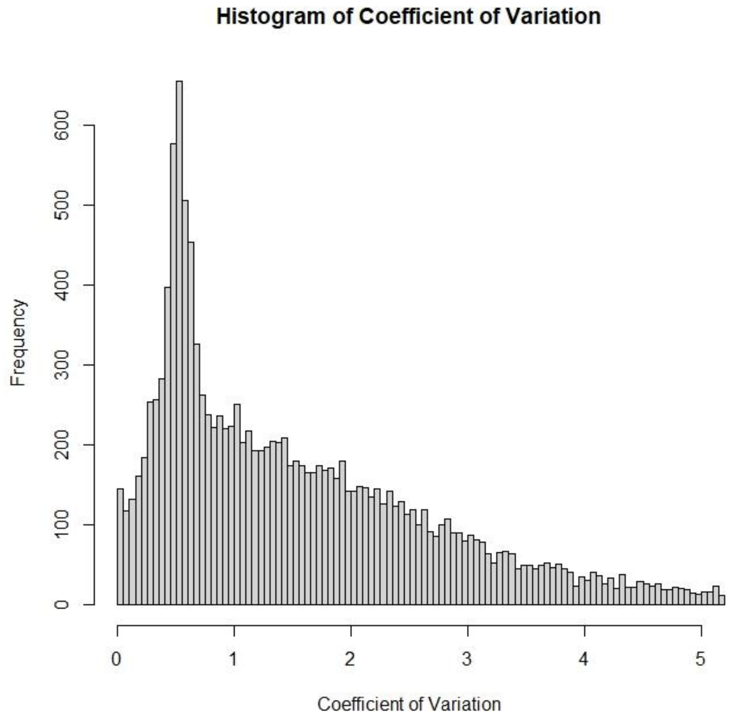

Supplementary Figure 2: Histogram of coefficient of variation. A coefficient of variation value was calculated for each gene. Genes with lower variation were filtered during the pre-processing step depending on the chosen threshold.

(A)

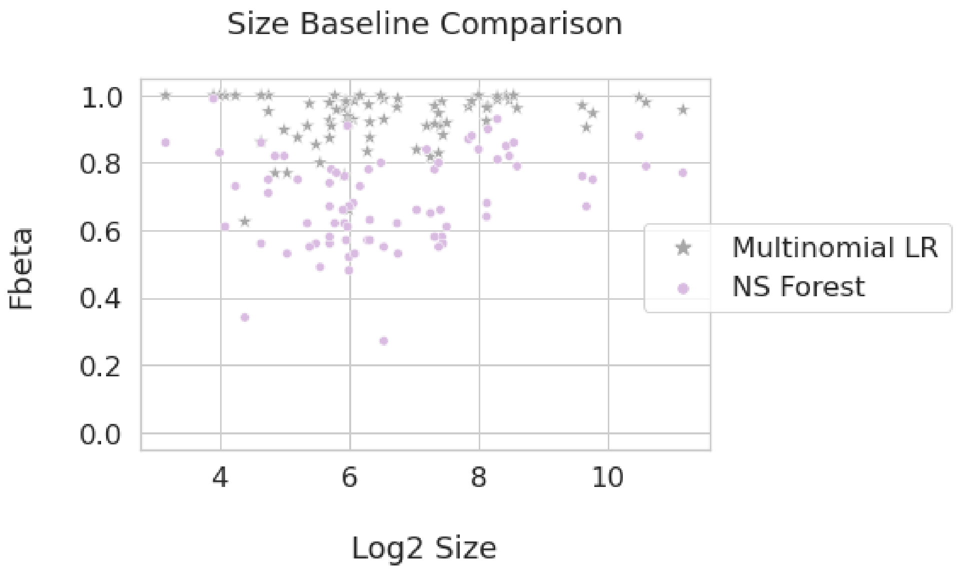

(B)

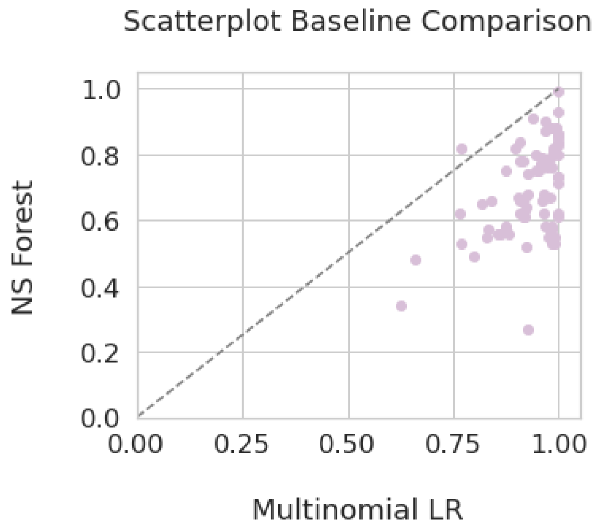

Supplementary Figure 3: Baseline Comparison between Multinomial Logistic Regression and NS Forest. F-beta distributions comparing the F-beta values between Multinomial Logistic Regression and NS Forest using optimal features for each algorithm and a 3.5 CV threshold.

Binary Logistic Regression (sklearn)	
penalty	l2
solver	saga
max_iter	100
multi_class	auto
n_jobs	-1
Multinomial Logistic Regression (sklearn)	
penalty	l2
solver	newton-cg
max_iter	100
multi_class	multinomial
n_jobs	-1
Support Vector Machine (sklearn)	
kernel	linear
regularization	1
Random Forest	
n_estimators	1200
Neural Network (Keras/Tensorflow)	
neurons per layer	100
number of hidden layers	3
activation function	relu
regularization	1e-6
optimizer	adam
loss function	Sparse Categorical Cross Entropy
LightGBM (LGBM)	
num_leaves	50
max_bin	50
min_data_in_leaf	1250
lambda_l2	1
extra_trees	TRUE
path_smooth	1

Supplementary Table 1: Optimal Hyperparameter Values. This table displays the optimal settings utilized for each machine learning method.

	lr	lr_multi	svm	rf	rf_multi	nn	lgbm
CV 0.52 CV 1.5	1.28E-06	1.52E-01	9.03E-09	NS	1.47E-01	8.43E-02	NS
CV 0.52 CV 2.5	3.13E-07	7.30E-04	1.89E-08	NS	1.90E-02	4.90E-10	9.46E-01
CV 0.52 CV 3.5	1.50E-04	5.75E-01	5.79E-07	NS	9.97E-05	2.61E-10	3.22E-02
CV 0.52 CV 4.5	1.75E-02	NS	8.20E-05	NS	3.46E-01	1.63E-09	5.44E-01
CV 1.5 CV 2.5	2.83E-04	1.16E+00	1.57E-02	NS	5.07E-01	7.92E-06	6.37E-01
CV 1.5 CV 3.5	2.53E-02	NS	1.33E+00	NS	4.21E-03	1.32E-07	4.84E-03
CV 1.5 CV 4.5	NS	NS	NS	NS	NS	2.92E-03	1.20E-01
CV 2.5 CV 3.5	NS	NS	NS	NS	5.55E-01	NS	1.09E+00
CV 2.5 CV 4.5	NS	5.16E-01	6.75E-01	NS	NS	NS	NS
CV 3.5 CV 4.5	1.56E+00	2.64E-01	3.00E-01	NS	1.07E+00	3.34E-01	NS

Supplementary Table 2: Wilcoxon P-Values For Various CV Thresholds. This table displays wilcoxon p-values for different CV thresholds of all machine learning methods utilizing the validation dataset.

	Default Train vs. Default Val	Optimal Train vs. Optimal Val	Default Val vs. Optimal Val	Optimal Val vs. Test
lr	3.50E-12	1.64E-12	0.795829538	0.920981475
lr_multi	1.10E-11	1.13E-13	1.13E-13	0.186445086
svm	3.55E-13	5.15E-12	0.001222993	0.807155438
rf	1.84E-14	1.84E-14	0.801590942	0.258408447
rf_multi	5.19E-13	2.40E-12	0.001373975	0.649697198
nn	5.21E-13	5.16E-12	2.53E-05	0.11984641
lgbm	1.13E-13	1.12E-12	5.28E-14	0.402887032

Supplementary Table 3: Wilcoxon P-Values for Default vs. Optimal Settings. This table displays comparisons between training, validation, and test datasets utilizing both default and optimal settings for each model.
