# Supplementary material for: Machine learning for cell type classification from single nucleus RNA sequencing data
Source: PLoS One. 2022 Sep 23;17(9):e0275070. doi: 10.1371/journal.pone.0275070 (PMC9506651; doi:10.1371/journal.pone.0275070)
Supplement: Attachment — Submitted filename: Response to reviewers_final.docx [file pone.0275070.s002.docx]

**Response to Reviewers**

Response overview:

We want to thank the reviewers for their valuable comments and revision suggestions for our manuscript. In response to the reviewers’ comments, we made the following major changes:

1. We added an independent analysis of an additional dataset, a kidney snRNA-seq dataset from the HuBMAP Consortium, to validate the machine learning model performance and test the robustness of the models with the optimal hyperparameters trained from the brain dataset. The evaluated models showed strong performance on the kidney data, with the multinomial logistic regression and LightGBM again being the top two performers, as observed with the brain (MTG) dataset. Interestingly, the optimal feature selection thresholds for coefficient of variation (CV) were slightly different for the two datasets, suggesting that heterogeneity exists in cell types between different tissues and customized informative feature selection strategies are needed for effective cell type classification in different single cell data.
2. We conducted a more complete literature review as suggested by the reviews, and relevant work is properly cited in the Introduction section, providing a more comprehensive landscape of previous studies.
3. We improved the figure quality; high resolution figures are uploaded with the revised manuscript.

By addressing the reviewers’ comments, we fell that the overall quality of our manuscript has been substantially improved and the methods evaluation made more rigorous. Below, please find our point-by-point response to reviewers’ comments.

Reviewer #1

1) The main purpose of the paper as they stated in the Introduction is the comparison of models and feature selection on the classification task. The type of biological data they analyzed (MTG of human brain) is irrelevant to this end. To have more robust conclusions from the modeling part they should apply the same procedure to another dataset.

- To address this comment, we added an independent analysis of an additional dataset, the kidney snRNA-seq dataset from the HuBMAP Consortium, to validate the machine learning model performance and test the robustness of the models with the optimal hyperparameters trained from the brain dataset. The evaluated models showed strong performance on the kidney data, with the multinomial logistic regression and LightGBM again being the top two performers, as observed with the brain data. Interestingly, the optimal feature selection thresholds for coefficient of variation (CV) were slightly different for the two datasets, suggesting that heterogeneity exists in cell types between different tissues and customized informative feature selection strategies are needed for effective cell type classification in different single cell data. A description of the new dataset, results, and model performance evaluation are discussed in highlighted text and Figure 6 on pages 8, 11, 12, 14.

2) Could be of interest to see how the 75 cell types are divided: are there cell types more represented than others? And if it is true, is the overrepresentation of a cell type correlated with the model performance?

- The composition of the 75 cell types in the human MTG cell type taxonomy can be found in Figure 1c in the original *Nature* publication of the brain dataset^1^.Some cell types are overrepresented and some are underrepresented and this is briefly mentioned in our Results section under Feature Selection and Hyperparameter Optimization on page 9 and Figure 3. Overall in all optimal models, cluster sizes between 2^4^ (16) and 2^8^ (256) showed more variability in performance suggesting that all methods performed worse when run on smaller cluster sizes, possibly due to fewer informative marker genes available for small clusters in the training data. Figure 4 shows model performance on the brain training, validation, and testing datasets in relation to the cell cluster sizes, a reflection of cell type prevalence in the original tissue.

3) They should describe in more details the hyperparameters tuning section, as it is a main point of the paper. In table 1 (that could also be moved in the supplementary) they just described what hyperparameters they would look at. There is no paragraph in the paper where they describe how they changed the parameter (for example, in the neural network model, how many hidden layers were tested? In the random forest, which number of trees were used?). In the end, how many models were trained?

- Hyperparameter tuning is described in the Hyperparameter Optimization section located on pages 9 and 10. For the sklearn models, we varied many model-specific parameters (for example kernels for SVM and penalty for Logistic Regression). This section now describes the ranges of hyperparameters tested and the general effect these changes had on the model; the full range of hyperparameters tested can be found on the Github repository with the specific link pointers provided in the manuscript on page 9. For example in the neural network results csv file, we tested 3 hidden layers. In the main body of the manuscript, Table 1, as part of the Methods section, we simply presented the results of optimal hyper-parameter settings, which would be of most use to the broad reader community of PLOS ONE who may not necessarily have the machine learning background. Having said that, the hyperparameter tuning results can be found in Supplementary Table 1 and the corresponding Github link.

4) The overfitting showed from the models is worrying. This is also a reason to test the approaches on another dataset. I would suggest evaluating the model on a cross-validation setting and if this does not change the outcome to try to justify the overfitting with the composition of the data (number of cells per cell type).

- Thank you for the reviewer’s suggestion. We performed an independent validation study using the new kidney snRNA-seq data. The kidney dataset, results and model performance evaluation are discussed in highlighted text and Figure 6 on pages 8, 11, 12, 14. Though some overfitting was observed in the model training stage of the brain dataset, the cell type classification models, including the hyperparameters, trained from the brain dataset showed strong performance on the independent testing kidney dataset (Fig. 6), which to certain extent addresses the robustness of the models and that the overfitting was only found in the training brain dataset, but not carried over to the validation and independent testing datasets. Due to the complexity of the real data, some rare cell populations in the highly granular cell type taxonomy will limit the computational feasibility of a cross-validation study. Using simulated or synthesized data with balanced cell type composition is a useful alternative strategy to overcome the limitations in real data, but the evaluation with respect to the cell type composition would go beyond the scope of this study.

5) The github page is missing the notebook for the neural network implementation. I would also suggest improving the readme with a bit of introduction and explanation of the folders.

- The Github repo has been updated with a “readme” file providing a better explanation of the folders and a brief explanation of our methods and files used . The neural network implementation has also been added. The link to the Github repository is at the beginning of page 8.

Reviewer #2

1. The literature is not well discussed. For example:

1.1 A similar benchmark study was recently published: Cao, X. et al. A Systematic Evaluation of Supervised Machine Learning Algorithms for Cell Phenotype Classification Using Single-Cell RNA Sequencing Data. Frontiers Genetics 13, 836798 (2022).

Authors should properly cite such related work and discuss the difference or the added contribution by their work.

- Thank you for pointing us to this relevant work. We have added a discussion of similar work in the Introduction section on page 3 and 4, and the relevant work is cited.

1.2 Besides general-purpose machine learning methods, there are other supervised learning methods tailored particularly for cell phenotype classification. For example, the following paper utilizes the proportion of dropout as a useful feature to help classification

Ji et al. scAnnotate: an automated cell type annotation tool for single-cell RNA-sequencing data. bioRxiv 1–9 (2022) doi:10.1101/2022.02.19.481159.

I suggest including a few methods like this in the evaluation, or at least properly discuss the existence of such related work.

- Thank you for pointing us to this relevant work. We have added a brief discussion of the work in the Introduction on page 4, and the relevant work is cited.

2. In the evaluation, authors selected F-beta score for beta=0.5, which is a quite subjective choice. I suggest authors evaluate performance using other beta values to see how beta value affect the results of evaluation.

- The choice of beta=0.5 was validated in a previous study^2^, where the effect of different beta values was objectively evaluated. We now added the citation immediately after the choice of beta=0.5 (on page 8) to navigate the interested readers to the previous study where different values of beta were evaluated.

3. The figure quality is bad. I can barely see what's in Figure 2,3,4. Authors need to provide higher resolution files and make the text in figure large enough to read.

- Figure quality has been addressed and high resolution figures are uploaded for the manuscript revision.

References:

1. Hodge, R.D., Bakken, T.E., Miller, J.A. *et al.* (2019). Conserved cell types with divergent features in human versus mouse cortex. Nature 573, 61–68. <https://doi.org/10.1038/s41586-019-1506-7>
2. Aevermann, B., Zhang, Y., Novotny, M., Keshk, M., Bakken, T., Miller, J., Hodge, R., Lelieveldt, B., Lein, E., & Scheuermann, R. H. (2021). A machine learning method for the discovery of minimum marker gene combinations for cell type identification from single-cell RNA sequencing. Genome research, 31(10), 1767–1780. <https://doi.org/10.1101/gr.275569.121>
