# Supplementary material for: Machine learning for cell type classification from single nucleus RNA sequencing data
Source: PLoS One. 2022 Sep 23;17(9):e0275070. doi: 10.1371/journal.pone.0275070 (PMC9506651; doi:10.1371/journal.pone.0275070)
Supplement: Attachment — Submitted filename: Response to reviewers_2nd revision_final.docx [file pone.0275070.s003.docx]

**Response to Reviewers**

The relevant changes made to the manuscript in response to the reviewers’ comments are underlined in the “Tracked Changes” version. A summary of the response to each reviewer concern is summarized in ***bold italic*** below.

Reviewer # 1

***No changes requested.***

Reviewer #2

Authors use one data set to compare multiple machine learning methods and found multinomial logistic regression work best.

There are two critical issues. I list them below

First, literature review is poorly done. Authors should know the field before they start this work. Those works should be properly cited and discuss authors new contributions added to existing work.

They should start with a review paper

1.Pasquini, G., Arias, J., Schäfer, P. & Busskamp, V. Automated methods for cell type annotation on scRNA-seq data. Comput Struct Biotechnology J 19, 961–969 (2021).

There are very comprehensive benchmark papers for specific cell type classfication, such as

2.Abdelaal, T. et al. A comparison of automatic cell identification methods for single-cell RNA sequencing data. Genome Biol 20, 194 (2019).

There are also benchmark paper for comparing machine learning methods on cell annotation using scRNA-seq data

3.Cao, X. et al. A Systematic Evaluation of Supervised Machine Learning Algorithms for Cell Phenotype Classification Using Single-Cell RNA Sequencing Data. Frontiers Genetics 13, 836798 (2022).

***We have expanded the Introduction section (page 3 – 4) to include a summary of these relevant publications and how our study differs. Also included in Literature citation***

Second, using one data set to claim one method is better is not comprehensive. Authors should change their statement to make it weak enough or use more data sets to check robustness of their comparison.1.

***We added the analysis of a second dataset derived from human kidney with a similar level of cellular complexity showing similar performance characteristics. This had been added as the new Figure 6 and described in the Methods (page 8), Results (page 11 – 12), and Discussion (page 14) sections.***
